# Using Wearable Devices to Monitor Activity and Sleep in Inpatients With Parkinson Disease With and Without Delirium: Feasibility and Acceptability Study

**DOI:** 10.2196/91009

**Published:** 2026-07-23

**Authors:** Gemma Louise Bate, Florence Gerakios, Alison Jane Yarnall, Sarah J Richardson, Laura Wright, John-Paul Taylor, David John Burn, Glenn Stebbins, Louise M Allan, Silvia Del Din, Rachael Ashleigh Lawson

**Affiliations:** 1 Translational and Clinical Research Institute, Newcastle University, 2nd Floor Biomedical Research Building, Campus for Ageing and Vitality, Newcastle upon Tyne, England, NE4 5PL, United Kingdom, 44 1912081277; 2 NIHR Newcastle Biomedical Research Centre, Newcastle Upon Tyne, England, United Kingdom; 3 Newcastle upon Tyne Hospitals NHS Foundation Trust, Newcastle upon Tyne, England, United Kingdom; 4 Rush University Medical Center, Chicago, IL, United States; 5 Centre for Research in Ageing and Cognitive Health, University of Exeter, Exeter, United Kingdom

**Keywords:** wearable devices, feasibility, acceptability, Parkinson disease, delirium, activity, sleep, inpatient, digital health

## Abstract

**Background:**

Delirium is a serious condition characterized by an acute change in attention, arousal, and sleep disturbance. It is common in inpatients with Parkinson disease (PD) but is frequently missed or misidentified due to overlapping symptoms, such as cognitive impairment, hallucinations, and sleep disturbances. While clinical tools often measure only a snapshot of delirium, wearable devices could facilitate the identification and ongoing monitoring of delirium, including continuous assessment of activity and sleep patterns, which are frequently disrupted. Establishing feasibility is essential before wearable technologies can be implemented in routine clinical care.

**Objective:**

This study aimed to determine the feasibility and acceptability of using wearable devices in inpatients with PD, with and without delirium.

**Methods:**

Participants were recruited from an ongoing prospective cohort study comprising inpatients with PD. Delirium was diagnosed using the *Diagnostic and Statistical Manual of Mental Disorders,* Fifth Edition (DSM-5) criteria and assessed daily. Participants were invited to wear Axivity AX6 devices attached to their lumbar region (lower back) and/or wrist for up to 7 days. Feasibility was assessed in terms of recruitment, device placement, nonsecurement, wear time, and compliance. Acceptability, practicality, and clinical constraints were recorded and compared between groups.

**Results:**

Participants were predominantly older adults with advanced PD and high levels of frailty and cognitive impairment. The wearable device recruitment rate was 75.4% (46/61), comprising 68 admissions. Delirium was identified in 64.7% (44/68) of admissions. The wrist-worn device showed greater participant acceptability, with 98.5% (67/68) of participants wearing a device on the wrist compared to 38.2% (26/68) of participants wearing a lumbar device (25/68, 36.8%, wore both). Wrist placement was more practical, rated as “somewhat” or “very easy” to secure in 95.5% (64/67) of cases compared to 69.2% (18/26) for lumbar placement (*P*<.001). Clinical constraints such as injury or pain and low level of arousal were associated with lumbar nonsecurement. These findings indicate that wrist-worn devices are more practical and acceptable in acutely unwell patients. Wear time compliance for both placements tended to be lower in delirium cases but comparable overall (>83% for each location, *P*>.05).

**Conclusions:**

This is the first study to evaluate the feasibility of using wearable devices in inpatients with PD with and without delirium. Wearable devices were feasible, and the wrist-worn devices demonstrated greater participant acceptability and practicality, with fewer clinical constraints. These findings provide important guidance for the design and implementation of future digital health studies in this population and may ultimately support earlier recognition and management of delirium while enabling continuous, objective monitoring of delirium-related changes not captured by standard clinical assessments.

## Introduction

Delirium is a serious, distressing neuropsychiatric syndrome defined by acute changes in attention, level of arousal, and cognition [1]. Other core features include motor fluctuations and sleep disruption [2]. People with Parkinson disease (PD) are at an increased risk of developing delirium compared to older adults (66% vs 38%), and this is associated with an increased risk of mortality, institutionalization, and dementia [3].

However, delirium in PD is underrecognized [4]. The overlap of presenting clinical symptoms between delirium and PD (in particular PD dementia [PDD]), such as fluctuations in attention and cognition, may partly explain this [5]. Simple bedside tests of attention and arousal can accurately detect delirium in individuals with PD and PDD [6,7]. However, clinical tools provide information for only a snapshot of time and do not capture symptom fluctuations central to delirium.

Wearable devices can monitor activity and sleep outcomes over a continuous period. This may have applications in delirium research and clinical management by reducing the need for repetitive assessments and capturing symptom fluctuations, such as overnight movement activity [8,9]. Intermittent clinical assessments may miss important changes in sleep and motor activity, such as day-night reversal, which can affect care and intervention strategies. Recent work has highlighted that changes in patterns of movement using actigraphy, including disrupted rest-activity cycles and altered motor behavior, may provide early indicators of delirium and support its detection using wearable technologies [9]. Furthermore, a recent study has demonstrated that wearable accelerometer data, combined with machine learning approaches, can be used to detect delirium episodes in participants poststroke [10].

However, there is a paucity of studies considering wearable devices in older adult inpatients with delirium [8], and none in delirium and PD [11]. A systematic review by Davoudi et al [8] identified 14 studies using wearable accelerometers in individuals with delirium, predominantly worn on the wrist or thigh. Of these, 6 studies characterized physical activity in inpatients with delirium, while only 3 evaluated sleep. Six studies used accelerometers to evaluate delirium or subtype detection; however, these were conducted in palliative care settings rather than in general inpatients. Overall, this review highlights that while wearable devices show promise for monitoring delirium, the evidence base remains limited, with few studies conducted in acute hospital settings and none in populations with PD.

A recent systematic review identified that wrist-worn devices are the most common and acceptable body placement for assessing activity and sleep in older adult inpatients [11]. The review found that 40 out of 89 studies placed a device on the wrist, reporting high tolerability and minimal invasiveness [11]. This may reflect their ease of use, familiarity (eg, similar to a wristwatch), and fewer clinical constraints compared to other body locations [12,13]. Wrist-worn devices can monitor sleep and circadian rhythms, as well as physical activity, including volume, intensity, and pattern. Recent findings have demonstrated that, to date, the wrist is the preferred site for obtaining sleep outcomes.

Most studies (70/89) used a single device, but 16 used more than 1 device, including those placed on the thigh, ankle, or chest [11]. While using multiple devices at different body positions could allow for more detailed quantification of postural transitions and gait, including core delirium features such as motor disturbances and mobility decline [11,14], these approaches may be less practical in acutely unwell inpatients. Importantly, mobility decline is associated with adverse outcomes, such as falls and functional deterioration [15]. Given that altered motor behavior is a core feature of delirium, single-site monitoring may still capture clinically meaningful changes in activity and functional status. To ensure interventions are effective, it is recommended that the feasibility and acceptability of a wearable device be among the first assessments conducted for the development of new digital health interventions [16]. This is particularly relevant as the demographic and clinical status of a patient can influence adherence to a wearable device [13].

This study aimed to evaluate the feasibility and acceptability of using wearable devices (Axivity AX6) attached to the lumbar region and wrist in inpatients with PD, with and without delirium. We hypothesized that the wrist-worn device would be more acceptable and practical to the participants than the lumbar attachment. Furthermore, we hypothesized that in cases with delirium, compliance would be poorer, with more frequent device removals and lower wear time compared to cases without delirium.

## Methods

### Population

Participants were recruited as part of a nested study within the “Defining Delirium and Its Impact in PD” (DELIRIUM-PD) study [3]. Participants were people diagnosed with PD who were admitted to the hospital between March 3, 2021, and January 6, 2022. Recruitment methods have been previously described [3]. In summary, written information was provided to potential participants with details to opt out of further contact. An electronic recurring admission patient alert was added to their hospital records, which alerted researchers of admissions to either the Royal Victoria Infirmary or Freeman Hospital, Newcastle upon Tyne, UK.

Exclusion criteria for DELIRIUM-PD comprised a diagnosis of nonidiopathic PD or atypical parkinsonian disorders or if they were not recruited within 72 hours of admission, lacked capacity to give informed consent and did not have an appropriate consultee available, were receiving end-of-life care, being isolated for infection control, being expected to be in the hospital for less than 24 hours, or having insufficient command of the English language to take part in the cognitive assessments. Additional participant exclusion criteria for this study included contraindications at both device attachment sites (left or right wrist and lumbar region), comprising pressure areas, broken skin, the presence of an intravenous cannula on the hand or wrist, or any other injuries. As this was a nested feasibility study within an existing cohort, no formal power calculation was performed; the study aimed to recruit approximately 50 admissions to provide sufficient data on recruitment, acceptability, compliance, and practical implementation.

### Data Collection

Baseline demographic and clinical data were collected at recruitment, including age, sex, PD duration (time from PD diagnosis by a movement disorders expert to admission), and preadmission cognitive impairment (as reported in the clinical records). Motor disease severity was assessed using the Movement Disorder Society Unified Parkinson Disease Rating Scale Part III [17] and Hoehn and Yahr stage [18]. The levodopa equivalent daily dose was calculated [19]. Baseline frailty was assessed using the Clinical Frailty Scale [20], and functional dependency was evaluated using the Schwab and England Activities of Daily Living scale [21].

Delirium was assessed prospectively based on the D*iagnostic and Statistical Manual of Mental Disorders,* Fifth Edition (*DSM-5*) criteria [1], as described by Richardson et al [22] (Multimedia Appendix 1). This assessment comprised bedside tests of attention and cognition, informant collateral history, and the assessor’s observations to establish an acute change from baseline. These structured assessments were conducted daily, in parallel with wearable device data collection, and served as the diagnostic reference standard for delirium status. In cases where there was diagnostic uncertainty, vignettes were presented to an expert consensus panel comprising 2 specialist independent reviewers and a third reviewer for any disagreements (FG and RAL, and AJY or SJR) [23].

### Device Placement

**Figure 1.**
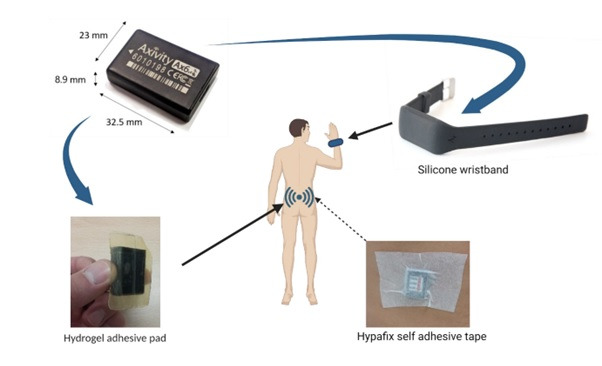
Wearable device setup for the wrist-worn and lumbar-placed device. Positioning of the Axivity AX6 wearable device (Axivity Ltd), secured to the wrist using a silicone wristband (Axivity Ltd; weight 16 g) and attached to the skin of the lumbar region using a hydrogel adhesive pad 1.1 mm thick, size 3.5 × 4.5 cm (Amgel Technologies), and secured with Hypafix dressing tape (BSN Medical Limited).

The Axivity AX6 wearable device (Axivity Ltd; dimensions: 23 × 32.5 × 8.9 mm; weight: 11 g) was worn continually during the study period, alongside daily delirium assessments, which were used as the diagnostic reference. The devices continuously recorded raw triaxial acceleration data, from which measures of physical activity, rest-activity patterns, and sleep-related metrics (eg, sleep duration and fragmentation) can be derived using validated processing approaches. The device was secured on the participant’s wrist most affected by their PD and/or on the lumbar region, unless contraindicated (Figure 1). Participants could opt to wear only 1 device (either on the wrist or lumbar region), and the device could be placed on the least affected wrist if determined by contraindications or participant/consultee preference. Contraindications included injuries, broken skin, and the presence of an intravenous cannula. If contraindications prevented placement on either wrist, the participants did not wear a wrist device.

The most affected wrist was ascertained by the Movement Disorders Society Unified Parkinson Disease Rating Scale Part III [17]. Where this was not possible, it was ascertained by asking participants/informants. The device on the lumbar region (L5) was secured to the skin by researchers using a hydrogel adhesive pad 1.1 mm thick, size 3.5 × 4.5 cm (Amgel Technologies) and secured with Hypafix (BSN Medical Limited; Figure 1). This method has been widely used in PD research within the community and is well tolerated [24,25]. A wristband (Axivity Ltd) was used to secure the device at the wrist. Participants were asked to wear the accelerometers continuously for up to 7 days during the admission period, including during normal activities, such as bathing/showering.

The clinical team was informed about the device by a patient alert flag on the participant’s hospital records and a bedside information sheet. This included the purpose of the devices, their location (left or right wrist and/or lumbar region), and advice to remove the device before diagnostic tests (eg, X-rays, computed tomography, and magnetic resonance imaging scans). Devices were checked during daily delirium reviews and, where appropriate, any removed devices were resecured.

### Feasibility Outcomes

The primary outcome measures were adapted from a conceptual framework of feasibility features [26]. They were defined as recruitment, acceptability, practicality, compliance, and preliminary feedback (Table 1). In addition to subjective feedback, objective measures of feasibility and acceptability were captured, including device wear duration, wear time as a percentage of the study period, and the frequency of device removals and resecurements. Device removal times were recorded based on participant and/or staff reports, where available. Where this was unclear (eg, due to cognitive impairment, delirium symptoms, staff changes, or lack of documentation), periods of nonwear were identified through visual inspection of the raw triaxial accelerometer data (eg, prolonged stationary signals).

**Table 1.** Definitions of the primary outcome measures.

Outcome	Measure
Recruitment	Percentage of participants from the DELIRIUM-PD^a^ study who wore one or more devices.
Acceptability	The number of cases that wore (1) a single wrist-worn device (no lumbar), (2) a single lumbar-placed device (no wrist), and (3) both wrist-worn and lumbar-placed devices. Clinical characteristics between groups.
Practicality	Researcher-rated scale, rating ease of device securement from 0 (“not possible”) to 5 (“very easy”).
Compliance	Number of days each device was worn (excluding any removal times). Wear time as a percentage of the study period (up to 7 days or until hospital discharge). The reasons and frequency of device removals/resecurements, including percentage of cases with devices secured at the end of the study period.
Preliminary feedback	Participant, carer, or staff feedback or concerns during each study visit.

a DELIRIUM-PD: Defining Delirium and its Impact in Parkinson disease.

### Data Analysis

All analyses were performed using SPSS Statistics (version 28; IBM Corp) with graphical visualization performed using Minitab (version 21.2). The normality of continuous variables was assessed by visual inspection of histograms and by using the Kolmogorov-Smirnov and Shapiro-Wilk tests of normality. Sample characteristics are provided as mean (SD), median (IQR), or frequency and percentage, as applicable. Independent-samples *t* tests, Mann-Whitney *U* tests, chi-square tests, and Fisher exact tests, as appropriate, were used to examine demographic and clinical differences between groups. Delirium was defined as meeting the DSM-5 criteria at any time during the repeated assessments for comparative analysis.

### Ethical Considerations

The DELIRIUM-PD study was approved by the Research Ethics Committee (Yorkshire & The Humber-Bradford Leeds Research Ethics Committee; 18/YH/0486) and conducted in accordance with the Declaration of Helsinki. No financial compensation or reimbursement was provided to participants for taking part in this study. Participants were approached as soon as possible after admission and provided with information about the study’s purpose and procedures. Potential participants were given time to ask questions and to consider their participation. If a participant lacked the capacity to provide full written informed consent, a personal consultee was identified to provide advice on participation, as per Section 32 of the Mental Capacity Act 2005 [27]. Data were pseudonymized and handled in accordance with ethical approvals and institutional research governance guidelines.

## Results

### Recruitment

**Figure 2.**
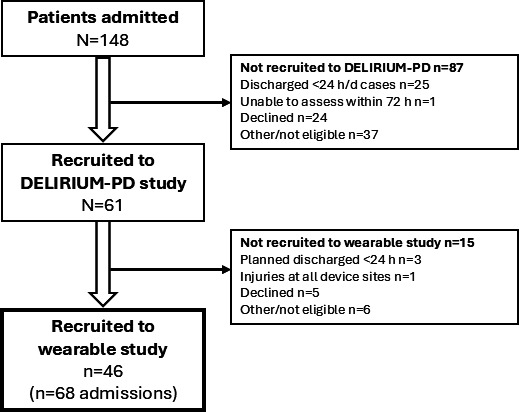
Recruitment flowchart. DELIRIUM-PD: Defining Delirium and Its Impact in Parkinson disease.

During the recruitment period, 148 patients with PD were admitted to the hospital (Figure 2). After exclusions, 61 (41.2%) participants were recruited to the DELIRIUM-PD study, and 75.4% (46/61) were recruited to this feasibility study (from 68 admissions). Six out of 61 (9.8%) participants were excluded from the study. Reasons for exclusion included a planned discharge in 3 participants (<24 h; 3/61, 4.9%), 1 participant who lacked capacity and did not have an appropriate consultee available, and 1 participant who had injuries at all device sites. Five out of 61 (8.2%) participants declined, consisting of 4 readmissions who had previously participated in the DELIRIUM-PD study but wanted to continue without any further component, and 1 participant who felt too unwell. This yielded an overall recruitment rate of 75.4% (46/61) from eligible participants.

### Participant Characteristics

Of the 68 admissions included in the study, 42 (61.8%) wore a device on the wrist only, and 26 (38.2%) wore a device placed on the lower back (lumbar region). Participants with baseline cognitive impairment, comprising mild cognitive impairment in PD (n=18) or PDD (n=9), more frequently wore a single device placed at the wrist compared to a lumbar placed device (50% vs 23.1%, respectively, χ²_1_=5.94.9; *P*=.027). No other clinical or demographic differences were found between those who wore the wrist and/or lumbar devices (*P*>.05 for all; Table 2). No differences were found between the recruited (46/61, 75.4%) and nonrecruited (15/61, 24.6%) participants for age, sex, PD duration, severity and stage, frailty, and baseline cognitive impairment (*P*>.05 for all; Multimedia Appendix 2).

**Table 2.** Demographics for the cases with a single wrist-worn device and cases with a lumbar placed device.

Characteristic	All (N=68)	Wrist only (n=42)	Lumbar^a^ (n=26)	Test statistic	*P* value
Continuous variables, median (IQR)					
Age (y)	78 (71.5-84)	77 (68.8-84)	81 (72.5-84)	500.5^b^	.56
Education (y)	11 (10-12.3)	11 (10-12)	10 (10-13.5)	484.5^b^	.69
MDS-UPDRS III^c^	54 (46-67)	53 (3-5)	58 (45-69)	358.0^b^	.72
Hoehn and Yahr stage	4 (3-5)	4 (4-5)	4 (3-5)	366.5^b^	.68
PD^d^ duration (y)	5.95 (3.7-9.9)	6 (3.1-13.5)	5.9 (3.9-7.2)	513.0^b^	.68
LEDD^e^ (mg/d)	600 (381.3-850)	650 (368.8-850)	525 (400-856)	529.5^b^	.84
Clinical Frailty Scale	6 (5-6)	6 (5-6.3)	6 (5-6)	523.0^b^	.76
GCS^f^ total	14 (13-15)	14 (11-15)	14 (13-15)	484.5^b^	..41
OSLA^g^ total	5 (3-8.5)	5 (3-11)	4 (3-7.3)	430.5^b^	.30
m-RASS^h^	0 (–1 to 0)	0 (–2.25 to 0)	0 (–1 to 1)	455.0^b^	.24
Schwab and England	50 (40-60)	50 (40-60)	50 (40-60)	483.5	.51
Categorical variables, n (%)					
Sex (female)	39 (57.4)	23 (45.2)	16 (61.5)	0.3 (1)^i^	.58
Cognitive impairment	27 (39.7)	21 (50)	6 (23.1)	4.9 (1)^i^	.027^j^
PD-MCI^k^	18 (26.5)	14 (33.3)	4 (16)	2.7 (1)^i^	.16k
PDD^l^	9 (13.2)	7 (16.7)	2 (7.7)	1.1 (1)^i^	.46^m^
Delirium	44 (64.7)	25 (59.5)	19 (73.1)	1.3 (1)^i^	.26

a Lumbar: n=25 lumbar + wrist, n=1 lumbar only.

b Mann-Whitney *U *test.

c MDS-UPDRS III: Movement Disorders Society Unified Parkinson's Disease Rating Scale Part III.

d PD: Parkinson disease.

e LEDD: Levodopa equivalent daily dose.

f GCS: Glasgow Coma Scale.

g OSLA: Observational Scale of Level of Arousal.

h m-RASS: modified Richmond Agitation and Sedation Scale.

i Chi-square test (*df*).

j Statistically significant results.

k PD-MCI: Mild Cognitive Impairment in Parkinson's disease.

l PDD: Parkinson disease dementia.

m Fisher exact test.

Delirium was identified in 64.7% (44/68) of cases. In both wrist-worn (n=67) and lumbar-worn (n=26) devices, cases with delirium were frailer (Clinical Frailty Scale: wrist, median 6, IQR 6-7 vs median 5, IQR 5-6; lumbar, median 6, IQR 6-6 vs median 5, IQR 4-5; *P*<.001, respectively), had more severe levels of arousal (Glasgow Coma Scale: wrist, median 14, IQR 11-14 vs median 15, IQR 15-15; lumbar, median 14, IQR 13-15 vs median 15, IQR 15-15; *P*<.05, respectively), and lower baseline independence (Schwab and England score: wrist, median 40, IQR 40-60 vs median 60, IQR 50-67.5; lumbar, median 40, IQR 40-50 vs median 60, IQR 50-40; *P*<.05, respectively) than those without delirium. In wrist-worn devices, cases with delirium were older (median 80, IQR 75-85 vs median 75, IQR 68-81.8; *P*<.05) than those without delirium (Multimedia Appendix 3).

### Acceptability and Practicality

A device was secured at the wrist in 98.5% (67/68) of admissions (Multimedia Appendix 4). This comprised a single wrist-worn device (no lumbar securement) in 61.8% (42/68) of admissions and combined wrist and lumbar devices in 36.8% (25/68) of admissions. A single lumbar device was secured in only 1 admission (1/68, 1.5%).

The lumbar device was not secured due to participant or consultee request in 50% (21/42) of cases, clinical factors (eg, injury and agitation) in 42.9% (18/42), staff unavailability in 4.8% (2/42), and informant request in 1 (2.4%) case. Clinical factors were more frequently cited as the reason for nonsecurement in those with delirium compared to those without delirium (72.7% vs 10.0% respectively; χ²_1_=16.8; *P*<.001; Table 3), primarily due to a low level of arousal and agitation or confusion (χ²_1_=4.019; *P=*.045 for both).

**Table 3.** Reasons provided for lumbar device nonsecurement for participants with and without delirium^a^.

Reasons	No lumbar device (n=42), n (%)	No delirium (n=20), n (%)	Delirium (n=22), n (%)	Chi-square (*df*)	*P* value
Acceptance factors	21 (50)	17 (85)	4 (18.2)	18.7 (1)	<.001^a^
Participant request	21 (50)	17 (85)	4 (18.2)	18.7 (1)	<.001^a^
Clinical factors	18 (42.9)	2 (10)	16 (72.7)	16.8 (1)	<.001^a^
Injury/pain	5 (11.9)	2 (10)	3 (14)	0.1 (1)	.72
Not responsive/low level of arousal	4 (9.5)	0	4 (18)	4.0 (1)	.045^a^
Agitation/confusion	4 (9.5)	0	4 (18)	4.0 (1)	.045^a^
Broken skin	3 (7.1)	0	3 (14)	2.9 (1)	.09
Connected to lines	2 (4.8)	0	2 (9)	1.9 (1)	>.99
Procedural factors	3 (7.1)	1 (5)	2 (9.1)	N/A^b^	N/A
Staffing (×2 needed)	2 (4.8)	1 (5)	1 (5)	0.0 (1)	>.99
Consultee/informant request	1 (2.4)	0	1 (5)	0.9 (1)	>.99

a Statistical significant results.

b N/A: not applicable.

No clinical constraints prevented the securement of the wrist-worn device. One participant with delirium did not wear the wrist device due to consultee request, as the participant disliked wearing a wristwatch. The wrist-worn device was secured on the least affected side in 19.4% (13/67) of the cases due to contraindications on the most affected side. This was most commonly due to cannula placement, which prevented securement in 14.9% (10/67) of cases. Broken skin, injury, and participant request prevented securement in 1.5% (1/67) case each.

Placing the wrist device was rated as “very easy” in 65.7% (44/67) of cases and “somewhat easy” in 29.9% (20/67) of cases. Placing the lumbar device was rated as “somewhat easy” in 57.7% (15/67) of cases and “somewhat difficult” in 19.2% (5/67) of cases. There were no missing data for any element of the analysis.

### Compliance

**Figure 3.**
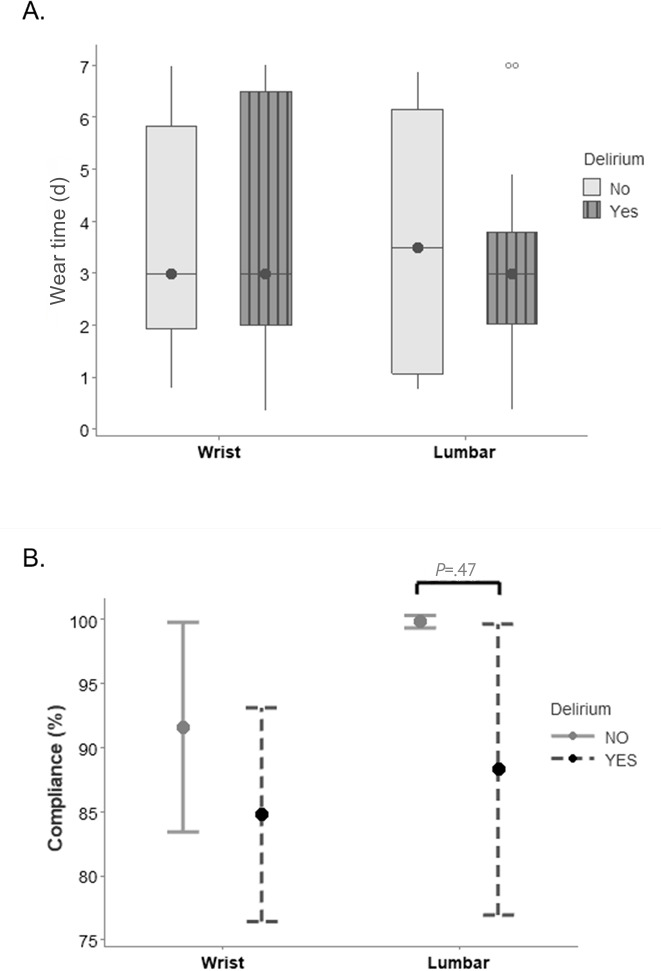
Comparison of (A) wear time (days), excluding wear time removal periods, and (B) wear time as a percentage of the study period (wear time compliance), presented as mean (SD), for the wrist and lumbar placed device for cases with and without delirium.

All measures of compliance were comparable for the wrist and lumbar devices (wear time: *P*=.43; Figure 3A; compliance: *P*=.41; Figure 3B). Lumbar wear-time compliance was lower in those with delirium compared to those without (mean 88.3%, SD 22.1% vs mean 99.8%, SD 0.5%, respectively; *P*=.047) and had a trend of being lower in wrist-worn devices with delirium than without (mean 83.9%, SD 27.0% vs mean 92.5%, SD 18.5%, respectively; *P=*.07; Figure 3B).

Early removals were comparable for cases with delirium for both wrist and lumbar positions (22/43, 51.2% vs 14/24, 58.3%; *P*=.45). In cases without delirium, the devices tended to be removed more often for the wrist device compared to the lumbar device (41.7% vs 14.3%; *P*=.18). Wrist-worn device removals were more frequently resecured in cases without delirium compared to those with delirium (8/10, 80% vs 7/21, 33.3%; *P*=.002). Of the 11 lumbar devices removed, 4 (36.4%) were resecured, and 75% (3/4) were resecured incorrectly. All wrist-worn device resecurements were correctly resecured.

### Reasons for Removals

The wrist-worn device was removed in 46.3% (31/67) of cases (mean removal occurrence 1.2, SD 0.6), most commonly due to self-removal (10/31, 32.3%) and hygiene purposes (6/31, 19.4%; Multimedia Appendix 5). The device placed on the lumbar region was removed in 42.3% (11/26) of cases (each consisting of a single removal), most commonly due to diagnostic purposes (3/11, 27.3%) and discomfort (2/11, 18.2%; Multimedia Appendix 5). Of the removal of lumbar devices, 90.9% (10/11) had delirium. Four devices were lost (2 wrist and 2 lumbar), all from cases with delirium (Multimedia Appendix 5). Slight skin irritation was found in 2 cases; 1 case showed slight reddening under the wristband, and 1 case had slight itching at the lumbar attachment site. Devices were immediately removed and not resecured.

### Participant Feedback

Feedback was provided in 76.1% (51/67) of cases with a wrist-worn device and 76.9% (20/26) of cases with a lumbar device. Among those who provided feedback, no concerns were reported or the device was described as comfortable in 94% (48/51) of cases with a wrist-worn device and 70% (14/20) of cases with a lumbar-placed device. Wearability concerns were expressed only in cases with delirium (lumbar: 6/20, 30%; wrist: 3/51, 5.9%). Concerns reported for lumbar-placed devices included pain (n=3) and attachment issues, such as sticking to bedsheets or clothing (n=3). Concerns reported for wrist-worn devices included it feeling “bulky and irritating,” the material being inappropriate if hot and sweaty, and the strap pulling at arm hairs (n=1 for each).

## Discussion

### Principal Results

This is the first feasibility study to evaluate lumbar and wrist-worn devices in inpatients with PD, with and without delirium. This study supports the use of wearable devices in the hospital setting and shows greater feasibility for the wrist-worn device. Using wearable technology in this vulnerable group of inpatients with PD provides an opportunity to measure activity and sleep patterns concurrently, which are features commonly disrupted in delirium.

### Comparison With Prior Work

This study has a relatively high recruitment rate, with three-quarters of eligible participants recruited [11]. The wrist-worn device had greater acceptability and practicality than the lumbar placement, with fewer clinical constraints and easier securement. The greater feasibility of wrist-worn devices may also reflect ergonomic advantages. Wrist placement is familiar (similar to a watch), minimally intrusive, and does not require repositioning of the patient, which is particularly important for acutely unwell inpatients or those with delirium [12,13]. In contrast, lumbar placement requires greater handling and may be less comfortable, likely contributing to lower acceptability. Lumbar devices have been reported as acceptable and comfortable mobility measures, used successfully in participants with PD to quantify real-world walking activity and gait [28-30]. However, we found a low acceptance rate. This could be explained by the participants in previous studies having early-stage PD, with Hoehn and Yahr stage II as opposed to stage IV in this study, and potentially reflecting the novel use of a delirium cohort. Additionally, there are likely fewer clinical contraindications in the home environment compared to the hospital setting, where the presence of injuries, broken skin, or an intravenous cannula can be problematic. As cognitive impairment is a risk factor for delirium [31] and a common feature of PD, it is essential that device placement is also feasible for this population. We found cases with cognitive impairment were more likely to wear a device at the wrist than the lumbar region, possibly due to familiarity [12,32] with the common placement of a wristwatch.

This is the first study to evaluate a lumbar device in inpatients with delirium. We found that low levels of arousal and agitation prevented lumbar securement in one-third of cases. Attachment of a device to the lower back generally involves more body movement to gain access to the lumbar region (generally by rolling onto a side or bending forward from sitting). With clinically unwell patients in the hospital setting, such maneuvers can be physically challenging for the participant, particularly in those with low levels of arousal and agitation. Consequently, a lumbar placement device may be more feasible in patients with less severe clinical symptoms. This was further supported in cases without delirium, which showed higher compliance (worn continuously without removal) for the lumbar placement in comparison to the wrist. Clinically, lumbar placement could also increase the risk of pressure areas, especially in patients with prolonged periods in bed with hypoactive delirium. This suggests that the wrist-worn device may be more acceptable for those with more severe hypoactive or hyperactive delirium [33]. However, for wearable monitoring to be clinically useful in delirium, device placement must be feasible for both patients with and without delirium.

Wrist-worn devices that were removed were more likely to be correctly repositioned in comparison to lumbar device removals. This was likely attributed to the greater practicality and ease of resecuring a wrist-worn device in clinically unwell patients, rather than the body positional changes generally required to resecure the device at the lumbar region. Unspecified self-removal and removal due to agitation were the most common reasons in admissions with delirium, while hygiene purposes were the most common in admissions without delirium. A previous study reported that devices were removed for bathing within the intensive care unit [34], and a further study using a single wrist device reported intolerance in 11.4% of participants [35]. However, specific reasons for removals are not always provided in inpatient wearable studies [11]; as a result, comparisons are limited. We found that removal due to adverse reactions was minimal, with minor skin irritation occurring in just 2 participants. Removal due to skin irritation has been reported in previous studies across a range of clinical populations including stroke [36], respiratory conditions [37,38], orthopedic surgery [39], oncology [40], and cardiology [41].

Overall, the wrist-worn device received more positive participant feedback. Wearability concerns occurred only in cases with delirium and occurred in over a quarter of cases with a lumbar placed device. Three participants with delirium reported pain at the site of lumbar device attachment, with the remaining cases reporting attachment or comfort concerns, whereas there were no reports of pain in those cases with a wrist-worn device. In addition to being feasible, we expect the wrist-worn device to provide important insights into sleep and physical activity, thereby supporting delirium detection in PD [42,43]. We anticipate digital outcome measures, such as wake after sleep onset, inactivity while awake, and daytime rest, which can offer frameworks on concepts like sleep fragmentation [11]. These findings also have potential implications for clinical care, where feasible wearable monitoring could support the detection and monitoring of delirium-related changes in activity and sleep patterns in routine practice. This may support earlier identification of delirium-related changes and complement bedside assessments that may miss its fluctuating nature, particularly as delirium is often underrecognized.

### Limitations

Strengths of this study were the use of robust delirium criteria based on the *DSM-5*, which were assessed prospectively. Conducting daily assessments in parallel ensured that the wearable devices were checked for proper positioning and adherence. Over the duration of the study, only 4 devices were lost, which is lower than previously reported [44]. There were several limitations in this study. First, the sample size was relatively small (n=68 admissions), with under half of the participants wearing the lumbar device. This makes it difficult to draw robust conclusions when assessing the influence of delirium on device compliance. However, this sample size is larger than that of most previous studies using wearables in delirium (79%), which ranged from 8 to 40 participants [8]. We aimed to approach all eligible patients within 72 hours; however, some patients were discharged before being approached for consent or were expected to stay <24 hours and thus were not included in the study. It is possible that patients with less serious health concerns (greater physical capacity) and less risk of delirium were underrepresented in this study. However, the cohort may more closely represent the clinical population at greatest risk of delirium due to more severe illness and longer hospital stay.

We did not include an older adult non-PD control group; therefore, the generalizability of these findings to other inpatient populations with delirium, such as older adults without PD, remains uncertain. Findings relating to device practicality and comfort may be applicable to other inpatient populations with delirium. However, PD-specific features, including motor impairment, cognitive fluctuations, and neuropsychiatric symptoms, may uniquely influence device acceptability and compliance. This should be considered for future studies. The time of removals of the devices were self-reported by participants or health care staff. Although we mitigated this with daily checks, gathered information from multiple sources, and visually checked these against the processed sensor data, these findings should be interpreted with caution. The study was completed during the COVID-19 pandemic, which reduced recruitment and may have influenced preference for device placement. Psychological factors influencing device removal (eg, discomfort, anxiety, or unfamiliarity) may be underreported in this cohort, particularly in those with delirium. Future studies incorporating qualitative methods, including interviews with patients, carers, and clinicians, will be important to better understand acceptability, perceived value, and implementation challenges in clinical settings.

### Conclusions

In summary, we found that the wrist-worn device had higher participant acceptance and greater practicality, with easier securement and fewer contraindications. We therefore recommend wrist-worn wearable devices as a feasible placement as a feasible location for future studies evaluating and establishing digital health outcomes of delirium in PD. These findings provide practical guidance for the design of future observational and interventional digital health studies, where device selection and placement should prioritize acceptability, minimize patient burden, and account for clinical factors such as delirium severity and mobility limitations. This may help standardize the reporting of digital outcomes in further studies while potentially providing important insights into activity and sleep metrics. Greater consistency between studies could facilitate the discovery of objective digital biomarkers for delirium detection, monitoring, and treatment response, including participant stratification and therapeutic end points in clinical intervention trials. In clinical practice, integrating wearable monitoring into care pathways may support more proactive and personalized management of delirium in hospitalized patients.

## Supplementary material

10.2196/91009 Multimedia Appendix 1 DSM-5 diagnostic criteria from the Delirium and Cognitive Impact in Dementia (DECIDE) study.

10.2196/91009 Multimedia Appendix 2 Clinical characteristics between recruited and not-recruited participants.

10.2196/91009 Multimedia Appendix 3 Characteristics of the cases with a wrist and lumbar-placed device, with and without delirium during the device wear time.

10.2196/91009 Multimedia Appendix 4 The device placement distribution for the recruited sample.

10.2196/91009 Multimedia Appendix 5 Device removal reasons and frequencies for the (A) wrist and (B) lumbar sensors.

## Abbreviations

DELIRIUM-PD: Defining Delirium and Its Impact in Parkinson disease
DSM-5: *Diagnostic and Statistical Manual of Mental Disorders,* Fifth Edition
PD: Parkinson disease
PDD: Parkinson disease dementia
