# Supplementary material for: Using Wearable Devices to Monitor Activity and Sleep in Inpatients With Parkinson Disease With and Without Delirium: Feasibility and Acceptability Study
Source: J Med Internet Res. 2026 Jul 23;28:e91009. doi: 10.2196/91009 (PMC13394853; doi:10.2196/91009)
Supplement: Multimedia Appendix 1 [file jmir-v28-e91009-s001.docx]

Supplementary Table 1: DSM-5 diagnostic criteria from the Delirium and Cognitive Impact in Dementia (DECIDE) study

| **DSM-5 criteria** | **Test to be performed or information needed** |
| --- | --- |
| **A.** Disturbance in attention (i.e., reduced ability to direct, focus, sustain, and shift attention) and awareness (reduced orientation to the environment) | Observations by the examiner during the interview (initiated by questioning such as “can you tell me what has been going on today?”)  Level of arousal measured using m-RASS and OSLA  Months of the year backwards  Digit Span from MDAS |
| **B.** The disturbance develops over a short period of time (usually hours to a few days), represents a change from baseline attention and awareness, and tends to fluctuate in severity during the course of a day | Acute onset and/or fluctuation obtained from informant history from nursing staff, next of kin and clinical notes |
| **C.** An additional disturbance in cognition (e.g., memory deficit, disorientation, language, visuospatial ability, or perception) | Impairment in any of the following domains:  SHORT-TERM MEMORY: three item recall at three minutes LONG-TERM MEMORY: when did World War II end?  ORIENTATION: 10 orientation questions from MDAS  LANGUAGE: 3 stage command, naming an object and explain purpose of object along with fluency, comprehension, and content of conversation  VISUOSPATIAL: Will a stone float on water?  PERCEPTUAL DISTURBANCE: evidence of illusions or hallucinations by collateral or direct observation/questioning |
| **D.** The disturbances in criteria A and C are not explained by another pre-existing, established, or evolving neurocognitive disorder and do not occur in the context of a severely reduced level of arousal, such as coma | Information from history/chart/clinical examination |
| **E.** There is evidence from the history, physical examination, or laboratory findings that the disturbance is a direct physiologic consequence of another medical condition, substance intoxication or withdrawal (i.e., because of a drug of abuse or to a medication), or exposure to a toxin or is because of multiple aetiologies | Information from history/chart/clinical examination |

Table as reported by (Richardson et al., 2017b). Abbreviations: m-RASS, Modified-Richmond Agitation and Sedation Scale; OSLA, Observational Scale of Level of Arousal; MDAS, Memoria Delirium Assessment Scale; DSM-5, Diagnostic and Statistical Manual of Mental Disorders, Fifth Edition.
