# Supplementary material for: Using Wearable Devices to Monitor Activity and Sleep in Inpatients With Parkinson Disease With and Without Delirium: Feasibility and Acceptability Study
Source: J Med Internet Res. 2026 Jul 23;28:e91009. doi: 10.2196/91009 (PMC13394853; doi:10.2196/91009)
Supplement: Multimedia Appendix 2 [file jmir-v28-e91009-s002.docx]

|  | **All**  **n=61** | | **Recruited**  **n = 46** | | **Not recruited**  **n = 15** | |  |  |
| --- | --- | --- | --- | --- | --- | --- | --- | --- |
| **Characteristic** | **Median (IQR)** | | **Median (IQR)** | | **Median (IQR)** | | **U/t** | **p-value** |
| Age years | 78 (9.0) | | 78 (9.5) | | 78 (7.0) | | 1353.0 | 0.946 |
| Education, y | 11 (2.0) | | 11 (2.1) | | 10 (1.0) | | 1326.5 | 0.128 |
| MDS-UPDRS III | 53 (25.0) | | 51 (22.8) | | 59 (29.0) | | 903.5 | 0.493 |
| Hoehn and Yahr stage | 4 (2.0) | | 4 (2.0) | | 5 (2.0) | | 901.0 | 0.454 |
| PD duration, y | 6 (6.9) | | 6 (6.1) | | 8 (8.8) | | 1315.0 | 0.431 |
| LEDD, mg/day | 550 (462.5) | | 575 (475.0) | | 475 (400.0) | | 1368.0 | 0.840 |
| Clinical Frailty Scale | 6 (2.0) | | 6 (2.0) | | 6 (1.0) | | 1333.5 | 0.639 |
| GCS total | 14 (2.0) | | 14 (2.3) | | 14 (1.0) | | 1381.0 | 0.635 |
| OSLA total | 5 (5.5) | | 5 (6.0) | | 5 (5.0) | | 1384.5 | 0.602 |
| m-RASS | 0 (2.0) | | 0 (2.0) | | 0 (2.0) | | 1337.0 | 0.701 |
| Schwab and England | 50 (20) | | 50 (20.0) | | 40 (40) | | 1275.5 | 0.892 |
|  | **n** | **%** | **n** | **%** | **n** | **%** | **ꭕ²** | **p-value** |
| Sex: male | 37 | 60.7 | 28 | 60.9 | 9 | 60.0 | 0.004 | 0.952 |
| Cognitive impairment | 29 | 47.5 | 19 | 41.3 | 10 | 16.7 | 3.414 | 0.181 |
| PD-MCI | 18 | *29.5* | 11 | 23.9 | 7 | 46.7 | 2.815 | 0.112^a^ |
| PDD | 11 | *18.0* | 8 | 17.4 | 3 | 20.0 | 0.052 | 1.000^a^ |
| Delirium | 32 | 52.5 | 26 | 56.5 | 6 | 40.0 | 1.429 | 0.232 |
| Delirium during admission | 41 | 67.2 | 32 | 69.6 | 9 | 60.0 | 0.460 | 0.537^a^ |

**Supplementary Table 2: Clinical characteristics between recruited and not-recruited participants**

Characteristics based on the initial recruitment visit. Abbreviations: GCS, Glasgow Coma Scale; LEDD, Levodopa equivalent daily dose; MDAS, Memorial Delirium Assessment Scale; MDS-UPDRS III, Movement Disorders Society Unified Parkinson's Disease Rating Scale Part III; m-RASS, modified Richmond Agitation and Sedation Scale; OSLA, Observational Scale of Level of Arousal; PD, Parkinson disease; PDD, Parkinson disease dementia; PD-MCI, Mild Cognitive Impairment in Parkinson disease; IQR, Interquartile range. a Fisher exact test.
