# Supplementary material for: Using Wearable Devices to Monitor Activity and Sleep in Inpatients With Parkinson Disease With and Without Delirium: Feasibility and Acceptability Study
Source: J Med Internet Res. 2026 Jul 23;28:e91009. doi: 10.2196/91009 (PMC13394853; doi:10.2196/91009)
Supplement: Multimedia Appendix 3 [file jmir-v28-e91009-s003.docx]

**Supplementary Table 3: Characteristics of the cases with a wrist and lumbar-placed device, with and without delirium during the device wear time**

|  | Wrist Device | | | | Lumbar Device | | | |
| --- | --- | --- | --- | --- | --- | --- | --- | --- |
|  | **No Delirium,**  **n=24** | **Delirium**  **n=43** |  |  | **No Delirium**  **n=7** | **Delirium**  **n=19** |  |  |
| Characteristics | **Median (IQR)** | **Median (IQR)** | **U/t** | **p value** | **Median (IQR)** | **Median (IQR)** | **U/t** | **p value** |
| Age | 75 (13.8) | 80 (10.0) | 650.5 | **0.031** | 75 (15.0) | 82 (10.0) | 76.0 | 0.296 |
| Education, y | 10 (1.75) | 11 (3.0) | 747.5 | 0.425 | 10 (2.0) | 11 (5.3) | 77.5 | 0.386 |
| MDS-UPDRS III | 51 (16.0) | 58 (25.5) | 585.0 | 0.243 | 48 (9.0) | 66(25.0) | 56.0 | 0.066 |
| Hoehn and Yahr stage | 3(1.0) | 3 (2.0) | 540.5 | 1.000 | 3 (1.0) | 4 (2.0) | 64.5 | 0.182 |
| PD duration, y | 6 (9.4) | 6 (5.6) | 902.0 | 0.263 | 6 (7.3) | 6 (2.8) | 97.5 | 0.885 |
| LEDD, mg/day | 840 (655.8) | 550 (400.0) | 890.5 | 0.333 | 840 (575.0) | 500 (375.0) | 98.0 | 0.862 |
| Clinical Frailty Scale | 5 (1.0) | 6(1.0) | 511.5 | **<0.001** | 5 (1.0) | 6(0.0) | 40.5 | **<0.001** |
| GCS total | 15 (0.0) | 14 (3.0) | 1159.0 | **<0.001** | 15 (0.0) | 14 (2.0) | 129.0 | **0.036** |
| OSLA total | 4 (2.0) | 7 (8.0) | 501.0 | **<0.001** | 3 (2.0) | 5 (5.0) | 47.5 | **0.006** |
| m-RASS | 0 (0.75) | -1 (4.0) | 896.0 | 0.285 | 0 (0.0) | 0 (3.0) | 98.5 | 0.835 |
| Schwab and England | 60 (17.5) | 40 (20.0) | 1037.0 | **0.001** | 60 (40.0) | 40 (10.0) | 144.0 | **0.004** |
|  | **n (%)** | **n (%)** | **ꭕ2** | **p value** | **n(%)** | **n(%)** | **ꭕ2** | **p value** |
| Sex: male | 12 (50.0) | 26 (60.5) | 0.687 | 0.407 | 3 (42.9) | 13 (68.4) | 1.412 | 0.369^a^ |
| Cognitive impairment | 7 (29.2) | 19 (44.2) | 1.463 | 0.226 | 6 (85.7) | 14 (73.7) | 0.417 | 1.000^a^ |
| *PD - MCI* | *7 (29.2)* | *11 (25.6)* | *0.101* |  | *6 (85.7)* | *12 (63.2)* | *0.021* |  |
| *PDD* | *0 (0.0)* | *8 (18.6)* | *5.07* |  | *0 (0.0)* | *2 (10.5)* | *0.798* |  |

*Note: Significant results are highlighted in bold. Abbreviations: GCS, Glasgow Coma Scale; LEDD, Levodopa equivalent daily dose; MDAS, Memorial Delirium Assessment Scale; MDS-UPDRS III, Movement Disorders Society Unified Parkinson's Disease Rating Scale Part III; m-RASS, modified Richmond Agitation and Sedation Scale; OSLA, Observational Scale of Level of Arousal; PD, Parkinson disease; PDD, Parkinson disease dementia; PD-MCI, Mild Cognitive Impairment in Parkinson disease; IQR, Interquartile range. a Fisher exact test*
