# Supplementary figures and images for: Using Wearable Devices to Monitor Activity and Sleep in Inpatients With Parkinson Disease With and Without Delirium: Feasibility and Acceptability Study
Source: J Med Internet Res. 2026 Jul 23;28:e91009. doi: 10.2196/91009 (PMC13394853; doi:10.2196/91009)

Supplementary Figure 1: The device placement distribution for the recruited sample


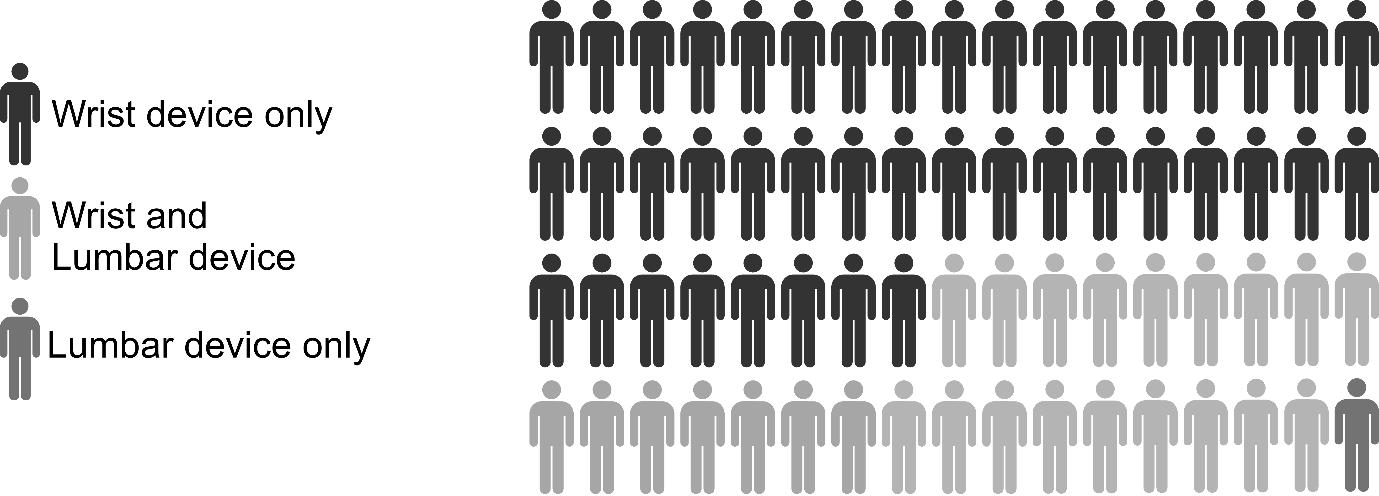

Supplement: Multimedia Appendix 4 [file jmir-v28-e91009-s004.docx]
