# Supplementary material for: Using Wearable Devices to Monitor Activity and Sleep in Inpatients With Parkinson Disease With and Without Delirium: Feasibility and Acceptability Study
Source: J Med Internet Res. 2026 Jul 23;28:e91009. doi: 10.2196/91009 (PMC13394853; doi:10.2196/91009)
Supplement: Multimedia Appendix 5 [file jmir-v28-e91009-s005.docx]

**Supplementary Figure 2: Device removal reasons and frequencies for the (A) wrist and (B) lumbar sensors.**


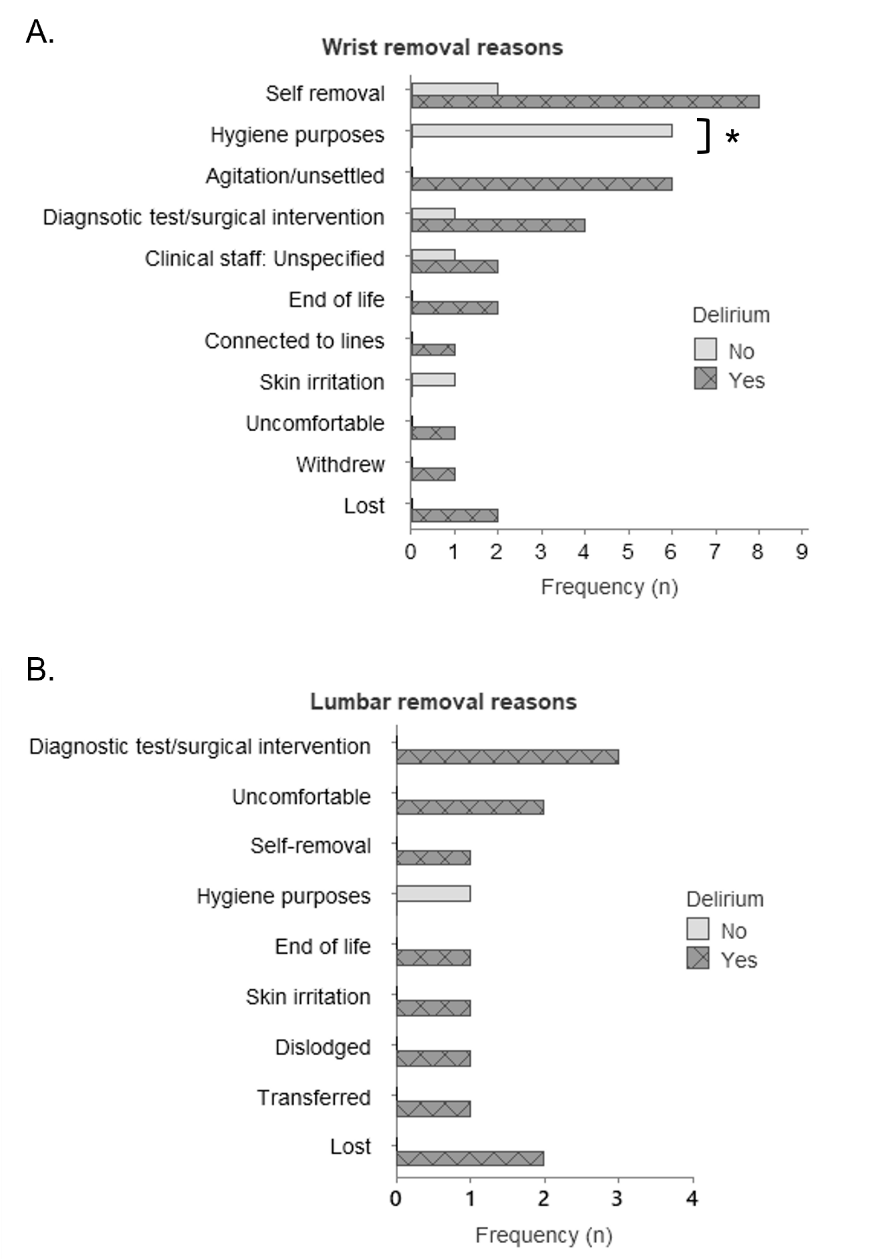


Symbols: *, significant difference in cases with and without delirium during the study period (p<.001).
